# Atypical Presentation of Acute Appendicitis with Schistosomiasis

**DOI:** 10.7759/cureus.15144

**Published:** 2021-05-20

**Authors:** Hassan A Alsayegh, Mohammed Q AlAlwan, Farhan Siddiqui, Qasem M AlAlwan, Haidar Alamer

**Affiliations:** 1 Department of Radiology, King Fahad Hospital, Hofuf, SAU; 2 Department of Pathology, King Fahad Hospital, Hofuf, SAU; 3 Department of Emergency Medicine, King Fahad Hospital, Hofuf, SAU

**Keywords:** schistosomiasis, appendicitis, computed tomography, histopathology

## Abstract

Schistosomiasis is a parasitic infection that is induced by different species of Schistosoma. The infection can manifest with a variety of different pathologies depending on the involved system and causative species. Schistosoma-induced appendicitis is rare in developed countries. We discuss a case of a middle-aged female who was diagnosed with acute appendicitis and underwent appendectomy. Upon histopathological examination of the resected appendix, Schistosoma infestation was identified as the underlying cause.

## Introduction

Schistosomiasis is a parasitic infection that is induced by different species of Schistosoma, either intestinal or urogenital. Intestinal species include the main species of blood fluke. Intestinal Schistosoma species include mansoni, japonicum, mekongi, geuineensis, and intercalatum [1]. It mostly affects rural and poor communities, particularly agricultural and fishing populations. Migration of population to urban areas is exposing the disease to new areas [1]. Schistosomiasis is a neglected tropical disease of poverty [2]. The primary transmission method is through contact with contaminated water sources [3]. It can be associated with poor personal hygiene and environmental sanitation. Infection occurs when larval forms of the parasite - released by freshwater snails - invade the skin during contact with infested water [1,4]. Review concerning the burden of the disease estimated that more than 200000 deaths per year are caused by schistosomiasis in sub-Saharan Africa [1]. 

Generally, appendicitis caused by Schistosoma is rare in developed countries like Japan, European countries, and the United States [3]. Schistosomiasis is considered an endemic disease in many areas, including the Eastern Mediterranean, some Indian ocean islands, the Arab peninsula, and the African continent. Tissue constriction, calcification, and fibrosis, especially of the urinary tract and appendix, are expected outcomes that can be caused by Schistosoma eggs. In endemic areas, Schistosoma haematobium eggs can be seen throughout the body, and these patients may complain of a variety of symptoms. Of note, Schistosoma eggs are often present in the appendix of these patients [5]. 

A 10-year Japanese study of 311 vermiform appendix pathologic archival specimens reported only a single case of schistosomal appendicitis [3]. This is in contrast with the prevalence of schistosomal appendicitis in endemic areas, such as sub-Saharan Africa, where cases of schistosomiasis are considered relatively high [2]. The importance of considering an infectious etiology for the induction of inflammatory processes in specific anatomical structures, such as the appendix, can be helpful and should be emphasized during history taking.

## Case presentation

**Figure 1:**
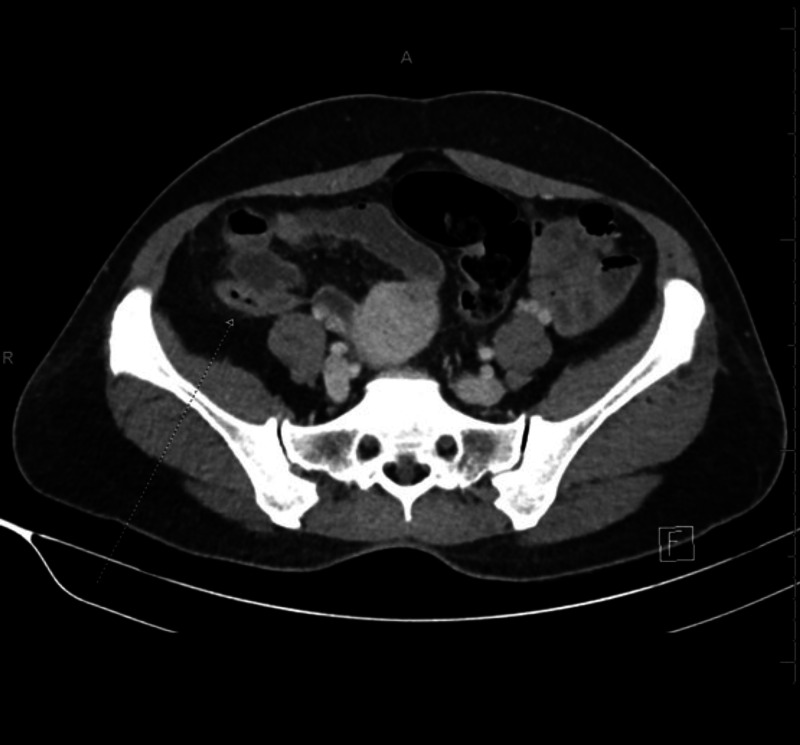
Axial enhanced computed tomography scan of the abdomen showing an inflamed appendix in the right iliac fossa (long arrow).

A 40-year-old Asian female was referred from a primary health care unit to our secondary hospital and trauma center. She complained of right iliac fossa pain that had started two days earlier and was progressive in nature. She had no other gastrointestinal symptoms. She reported that her urine had become darker than usual and accompanied the development of her abdominal pain. On physical examination, her vital signs were stable. She was conscious and oriented but in severe pain. On abdominal examination, there was right lower quadrant tenderness with rebound tenderness and a positive Rovsing’s sign. Laboratory tests were unremarkable except for a white blood cell count of 15.08 x 10^9^/L. Enhanced computed tomography revealed a right iliac fossa appendix that was inflamed and dilated in caliber with wall thickening and enhancement but no intramural appendicolith (Figure 1). Laparoscopic appendectomy was performed. The appendix specimen measured 6.2 cm x 2.7 cm with a dull outer surface and lumen showing brownish material.

The patient was given praziquantel in addition to surgery and observed for one day and discharged home in satisfactory condition.

**Figure 2:**
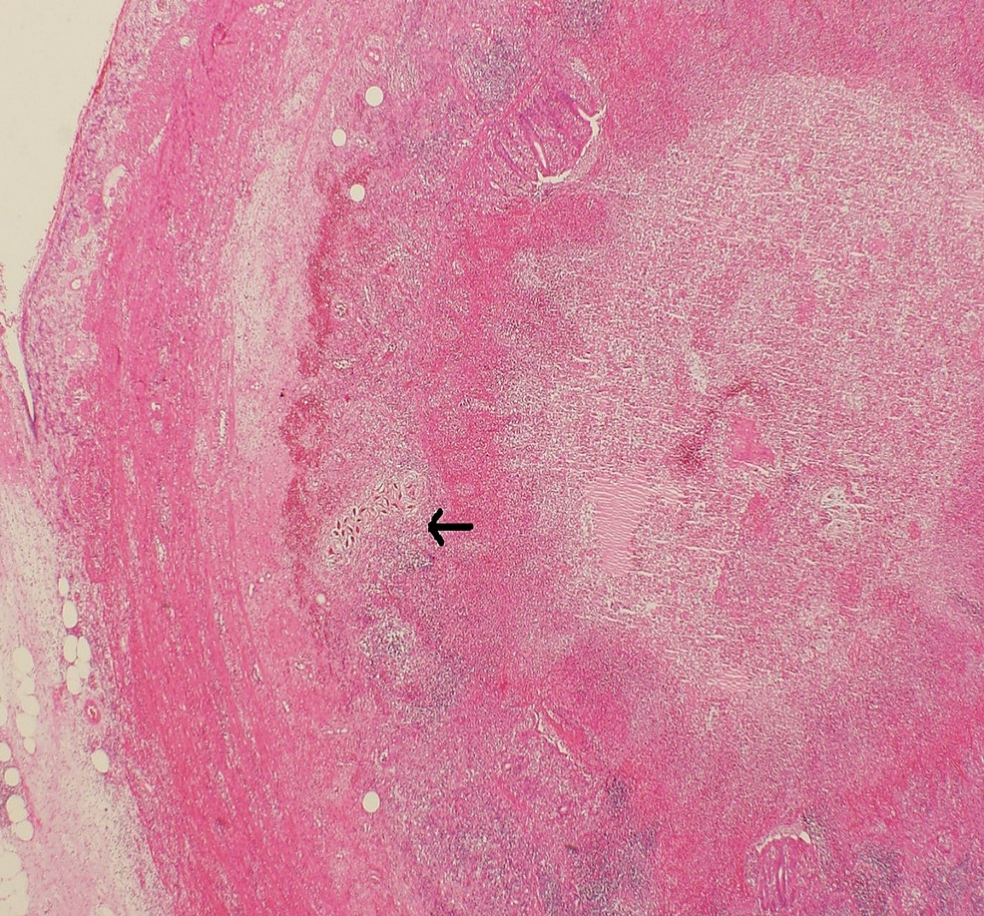
Photomicrograph showing appendiceal tissue with dense suppurative inflammation (arrow) (hematoxylin and eosin stain, 20x).

**Figure 3:**
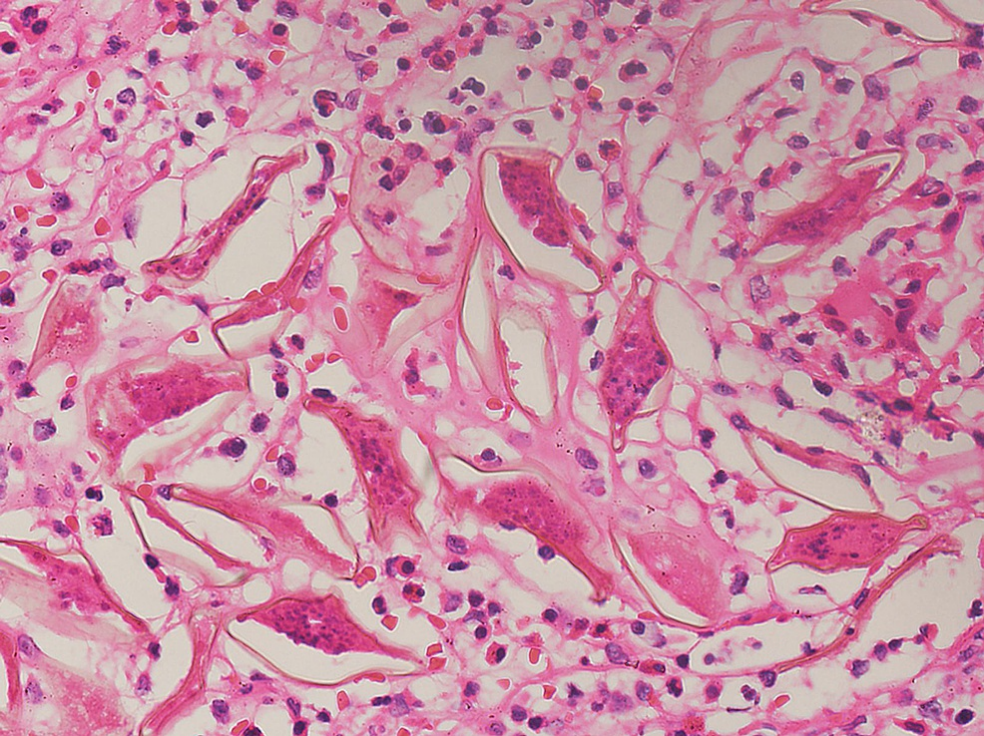
Photomicrograph showing viable Schistosoma eggs along with dense suppurative inflammation comprised of many eosinophils and neutrophils (hematoxylin and eosin stain, 400x).

Histopathological examination showed appendiceal tissue with dense suppurative inflammation comprised of many eosinophils and neutrophils along with clusters of Schistosoma eggs (Figures 2-3).

Upon retrospective questioning, the patient admitted that she used to swim and utilize the river water that is found in her village in her home country when she was younger, which explains the presence of Schistosoma eggs in the microscopic examination findings.

## Discussion

This case report presents a patient who was managed in a regional governmental hospital and trauma center that serves a large geographic region with a range of socioeconomic levels. People of different nationalities live in this region, as it is located around active economic, educational, and industrial institutions.

In 1909, Turner first described appendicular schistosomiasis [6]. A high prevalence of schistosomiasis in Saudi Arabia was reported by Bolbol and Mahmoud [7]. Some important Schistosoma species that have wide geographic distribution have been described, such as S. haematobium, S. mansoni, and S. japonicum. These species can deposit eggs into the appendix. However, appendicitis is rarely induced by these species [8-10]. Schistosoma-induced appendicitis is rare in developed countries like Japan and the United States, where the incidence is around 0.2% and 0.34%, respectively [11-13]. Conversely, appendicitis caused by Schistosoma is seen more often in the developing world. For example, a Nigerian study explored the histopathological examinations of multiple organs, including the appendix. Autopsies of 34 females and 54 males revealed positive results for Schistosoma in bladder tissue in 37% of samples. Further examination exhibited Schistosoma eggs in the appendix, and the severity of the disease increased with total incidence [14]. Schistosomiasis is acquired by exposure to contaminated freshwater.

This case report presents a patient who was managed in a regional governmental hospital and trauma center that serves a large geographic region with a range of socioeconomic levels. People of different nationalities live in this region, as it is located around active economic, educational, and industrial institutions.

In our case, the 40-year-old female patient's last visit to her home country the Philippines was five years ago. The Philippines is known to have endemic Schistosomiasis. However, the 40-year-old female patient presented with typical signs and symptoms of acute appendicitis. Computed tomography results cannot differentiate acute appendicitis caused by Schistosoma species from other etiologies. Microscopic examination is considered the gold standard to diagnose schistosomal ova in urine or stool. Mild infections can be detected by serological examinations [15]. Various studies have reported different microscopic findings of appendicitis caused by Schistosoma, such as reactive lymphoid hyperplasia [16], inflamed appendix with numerous submucosal Schistosoma that were shown to be mineralized [14], and suppurative inflammation with multiple schistosomal ova [17]. In contrast, the present case showed schistosomal eggs along with dense suppurative inflammation comprised of neutrophils and eosinophils, which has also been found in other studies.

## Conclusions

Acute appendicitis is very common and identifying its etiology is challenging. Radiological studies can support the diagnosis but cannot differentiate the etiology. Histopathological studies are required to identify the exact etiology. Surgical management is the standard management of acute appendicitis in most instances. Schistosoma-induced appendicitis should be considered when dealing with patients presenting from endemic countries. In addition to surgery, praziquantel is recommended in similar cases.
